# Unique Suites of Trabecular Bone Features Characterize Locomotor Behavior in Human and Non-Human Anthropoid Primates

**DOI:** 10.1371/journal.pone.0041037

**Published:** 2012-07-18

**Authors:** Timothy M. Ryan, Colin N. Shaw

**Affiliations:** 1 Department of Anthropology, Pennsylvania State University, University Park, Pennsylvania, United States of America; 2 Center for Quantitative Imaging, EMS Energy Institute, Pennsylvania State University, University Park, Pennsylvania, United States of America; 3 PAVE Research Group and The McDonald Institute for Archaeological Research, Department of Archaeology and Anthropology, University of Cambridge, Cambridge, United Kingdom; Illinois State University, United States of America

## Abstract

Understanding the mechanically-mediated response of trabecular bone to locomotion-specific loading patterns would be of great benefit to comparative mammalian evolutionary morphology. Unfortunately, assessments of the correspondence between *individual* trabecular bone features and inferred behavior patterns have failed to reveal a strong locomotion-specific signal. This study assesses the relationship between inferred locomotor activity and a *suite* of trabecular bone structural features that characterize bone architecture. High-resolution computed tomography images were collected from the humeral and femoral heads of 115 individuals from eight anthropoid primate genera (*Alouatta, Homo, Macaca, Pan, Papio, Pongo, Trachypithecus, Symphalangus*). Discriminant function analyses reveal that subarticular trabecular bone in the femoral and humeral heads is significantly different among most locomotor groups. The results indicate that when a suite of femoral head trabecular features is considered, trabecular number and connectivity density, together with fabric anisotropy and the relative proportion of rods and plates, differentiate locomotor groups reasonably well. A similar, yet weaker, relationship is also evident in the trabecular architecture of the humeral head. The application of this multivariate approach to analyses of trabecular bone morphology in recent and fossil primates may enhance our ability to reconstruct locomotor behavior in the fossil record.

## Introduction

Trabecular bone plays a significant structural role in the skeletal system [1]–[10] and has been shown to respond to the loading environment throughout ontogeny [10]–[13]. Despite its clearly mechanical function, attempts to identify locomotion-specific architectural characteristics in the postcranial trabeculae of primates have produced largely mixed results [11], [13]–[31]. While a few studies have found structural differences apparently related to divergent locomotor loading patterns [17], [19], [26], others have failed to detect significantly different structural characteristics across groups [16], [20], [24], [32]. Variation in data collection methods, quantification procedures, sample selection, sample size, anatomical location, and volume of interest (VOI) selection may all influence the detection of a ‘locomotor signal’ in trabecular bone. The fundamental question of whether trabecular bone architecture in complex postcranial joints, such as the proximal femur and humerus, reflects a strong locomotion-specific signal remains unresolved. Demonstration of a strong functional signal within trabecular bone would aid reconstructions of locomotor behaviors in the fossil and archaeological record.

Shaw and Ryan [32] recently compared subarticular humeral and femoral head trabecular bone morphology as well as humeral and femoral mid-diaphysis cortical bone structure among eight anthropoid genera. In contrast to comparisons of inter-limb diaphyseal bone robusticity, which display a strong locomotor signal [33], femoral head trabecular bone was significantly more robust (higher bone volume fraction, lower trabecular spacing) than humeral head trabecular bone in all taxa. Comparisons revealed an osteological ‘locomotor signal’ indicative of differential use of the forelimb and hind limb in diaphyseal cortical bone geometry, but not in subarticular trabecular bone.

Analyses of interspecific variation using single morphometric variables alone (e.g., bone volume fraction, trabecular number, degree of anisotropy) may be inadequate for identifying morphological patterns that reflect adaptation to habitual locomotor loading. Studies addressing the interrelationships among trabecular bone features have demonstrated a strong correlation between the various morphometric variables and bone volume fraction [2], [22], [34]. Mittra et al. [2], [34], using samples from sheep and humans, found strong relationships between bone volume fraction and trabecular thickness, spacing, number, connectivity, and structure model index, a measure of the relative proportion of plate-like and rod-like trabeculae. Cotter et al. [22] found similar relationships in the vertebral bodies of apes and humans with the notable exceptions of trabecular thickness and degree of anisotropy, neither of which correlated significantly with bone volume fraction in their sample. The results from these studies suggest that as bone volume fraction increases, trabeculae become more numerous, more plate-like, less widely spaced, and more interconnected. The strength of the interrelationships among other structural features varies, but is generally less robust. Trabecular thickness and anisotropy do not correlate strongly with many other variables, aside from bone volume fraction and structure model index, while features such as trabecular number, connectivity, and spacing display strong correlations with each other. Considering these structural relationships, analyses that account for variation within an entire suite of trabecular bone properties (multiple architectural variables) might be more appropriate, and indeed may prove more accurate for identifying locomotor and functional signals.

**Table 1:** Attributes of the taxonomic sample used in the current study.

Genus	Species	Museum	Locomotor Category	Demographics	Estimated Body Mass (kg)
Alouatta	*caraya*	AMNH	arboreal quadruped, climber	M: 4, F: 9	5.79 (0.96)^a^
***Homo***	*sapiens*	PSU	biped	M: 10, F: 10	60.86 (6.41) b
*Macaca*	*fascicularis*	MCZ	arboreal quadruped	I:19	4.07 (0.92) c
*Pan*	*troglodytes, verus,* *schweinfurthii*	AMNH	terrestrial quadruped, climber	M: 11, F: 4, I: 2	50.13 (10.22) d
*Papio*	*anubis, cynocephalus,* *hamadryas, ursinus*	AMNH,NMNH	terrestrial quadruped	M: 2, F: 4, I: 5	18.25 (4.72) c
*Pongo*	*pygmaeus, abelii*	NMNH	quadrumanous, climber	M: 5, F: 2	65.70 (21.50) e
*Trachypithecus*	*cristatus*	MCZ	arboreal quadruped	I: 21	5.92 (0.80) f
*Symphalangus*	*syndactylus*	NMNH	brachiator	M: 3, F: 4	10.77 (2.48) e

Length and body mass data presented as: mean (standard deviation).

NMNH: National Museum of Natural History (Smithsonian Museum), Washington, USA; American Museum of Natural History, New York, USA; PSU: Norris Farms Collection, Pennsylvania State University, Department of Anthropology, MCZ: Museum of Comparative Zoology, Harvard University.

M: Male, F: Female, I: Indeterminate.

Body Mass Estimation Equations:

a Haplorhine: 2.729*LN(FemHeadSI)+1.42) (SEE  = 0.239) [66].

b Female: (2.426*FemHeadAP-35.1)*0.9 (SEE  = 17.5); Male: (2.741*FemHeadAP-54.9)*0.9 (SEE  = 13.7) [68].

c Cercopithecine: (2.389*LN(FemHeadSI)-4.541))*1.014 (SEE  = 0.1670) [67].

d All hominoids: (3.019*LN(FemHeadSI)-6.668))*1.006 (SEE  = 0.1137) [67].

e Asian ape: (3.024*LN(FemHeadSI)-6.718))*1.008 (SEE  = 0.1309) [67].

f Colobines: (2.424*LN(FemHeadSI)-4.684))*1.01 (SEE  = 0.1385) [67].

By partitioning trabecular bone architecture into its component parts and focusing on pairwise comparisons among taxa, previous studies may have inadvertently atomized the complex inter-dependent structure of trabecular bone and consequently precluded identification of a relevant locomotor signal. This approach of examining individual morphological features is appropriate for analyses of cortical bone cross-sectional geometry, for example, because each measureable cortical bone feature (i.e. cortical area, torsional rigidity) has a direct and well-understood biomechanical implication [35]–[41]. A more accommodating paradigm for analyses of trabecular bone architecture may be to apply the principle of *Holism*, and consider whether ‘the whole is greater than the sum of its parts’ [42].

**Figure 1:**
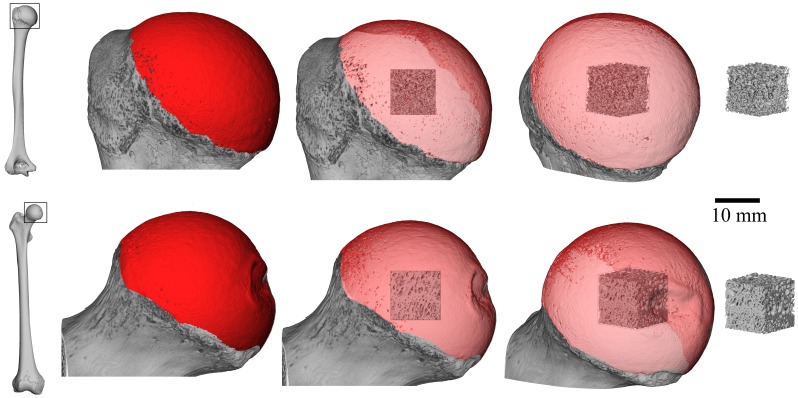
Volume of interest (VOI) selection method. The articular surface of the humeral and femoral heads (shown in red) were extracted from a three-dimensional isosurface reconstruction. The volume of interest was scaled based on the size of a best-fit cube for the articular surface and was positioned in the center of the humeral or femoral head.

The goal of this study is to assess whether variation in features of subarticular trabecular bone morphology, taken as a collective suite, accurately reflects differences in locomotor patterns across anthropoid primate taxa. This question is addressed using eight anthropoid genera that can be coarsely differentiated into separate locomotor categories. The first hypothesis is that groups with more derived locomotor patterns such as bipeds (*Homo*) and brachiators (*Symphalangus*) will express trabecular bone patterns that are significantly different from one another, while groups with more similar locomotor patterns, such as arboreal quadrupeds (*Alouatta, Macaca, Trachypithecus*) and terrestrial quadrupedal climbers (*Pan*), will express more similar trabecular bone architecture. Secondly, because the primate forelimb is used less extensively for propulsion during locomotion and is often used to perform more diverse manipulative behaviors, it is hypothesized that humeral head trabecular architecture will display less variation among locomotor groups.

**Table 2:** K-statistic calculated for select femoral head and humeral head trabecular bone measurements.

Variable	K
Fem Conn.D	0.2614
Fem SMI	0.4219
Fem Tb.N	0.5308
Fem Tb.Th	0.5033
Fem Tb.Sp	0.5234
Fem DA	0.2928
Hum Conn.D	0.4121
Hum SMI	0.2987
Hum Tb.N	0.5551
Hum Tb.Th	0.5933
Hum Tb.Sp	0.5479
Hum DA	0.4416

## Materials and Methods

### Sample

The skeletal sample used in the current study consisted of one femur and one humerus from a total of 115 individuals from eight anthropoid genera (Table 1). All non-human specimens were wild-shot adults and exhibited no external signs of pathology or trauma. Age at death was estimated only for *Homo*. Individuals who displayed external signs of osteological senescence (i.e. osteoarthritis, eburnation) were excluded from the study. Bones from both right and left sides were used in the sample, one femur and humerus per specimen, but only elements from the same side were used for a single individual.

**Figure 2:**
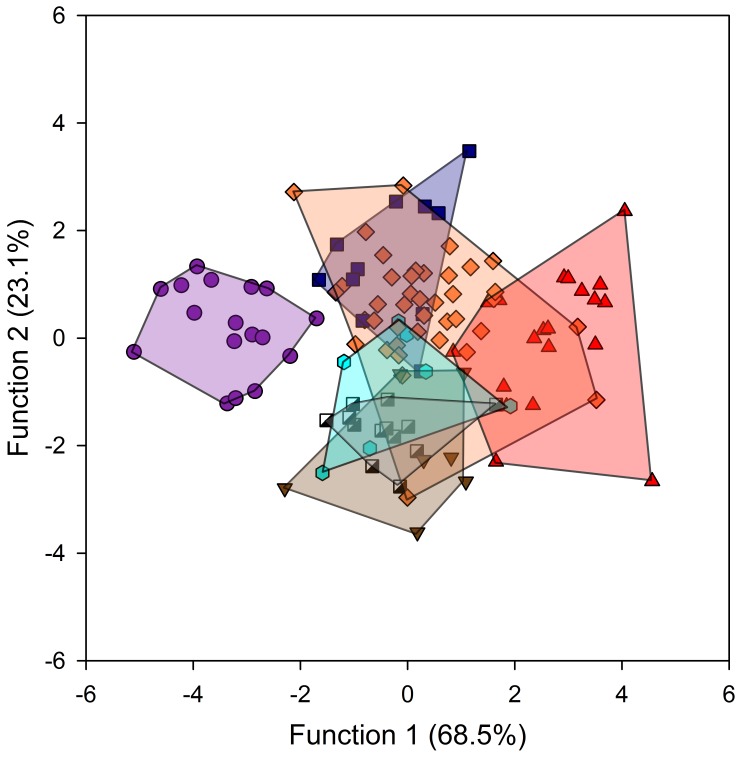
Results of multivariate discriminant function analyses for the femoral head. Symbols: red triangles, bipeds; blue squares, terrestrial quadrupeds; orange diamonds, arboreal quadrupeds; black and white squares, arboreal quadruped-climbers; cyan hexagons, quadrumanous climbers; brown triangles, brachiators; purple circles, terrestrial quadruped-climbers.

**Table 3:** Stepwise discriminant function analysis for locomotor group using five femoral head trabecular bone variables.

		Predicted group membership							
Locomotorgroup	n	Biped	Terrestrial quad	Arboreal quad	Arboreal quad-climber	Quadrumanous climber	Brachiator	Terrestrial quad-climber	%correct
Biped	20	17	0	2	1	0	0	0	85.0
Terrestrial quad	11	0	8	1	1	1	0	0	72.7
Arboreal quad	40	4	8	22	1	5	0	0	55
Arboreal quad-climber	13	0	0	0	12	0	1	0	92.3
Quadrumanous, climber	7	1	0	1	1	4	0	0	57.1
Brachiator	7	0	0	1	0	1	5	0	71.4
Terrestrial quad- climber	17	0	0	0	0	0	0	17	100.0

Classification results (total correct  = 73.9%).

Biped: *Homo*; Terrestrial quadruped: *Papio*; Arboreal quadruped: *Macaca, Trachypithecus*; Arboreal quadruped-climber: *Alouatta*; Quadrumanous climber: *Pongo*; Brachiator: *Symphalangus*; Terrestrial quadruped-climber: *Pan*.

**Table 4:** Pooled within-group correlations (r) between functions and variables.

Variable	Function 1	Function 2
Conn.D	0.024	0.256
SMI	0.329	−0.160
Tb.N	−0.374	0.802
Tb.Th	−0.136	−0.003
DA	0.761	0.569

**Table 5:** F-test results between groups (DF = 3, 106) for stepwise discriminant function analyses for locomotor group using five femoral head trabecular bone variables.

	Biped	Terrestrialquad	Arboreal quad	Arboreal quad-climber	Quadrumanous climber	Brachiator	Terrestrial quad-climber
Biped	**X**	<0.001	<0.001	<0.001	<0.001	<0.001	<0.001
Terrestrial quad	22.73	**X**	NS	<0.001	<0.001	<0.001	<0.001
Arboreal quad	25.02	2.58	**X**	<0.001	<0.01	<0.001	<0.001
Arboreal quad-climber	31.16	20.23	19.58	**X**	NS	<0.01	<0.001
Quadrumanous, climber	14.30	8.33	5.46	2.00	**X**	NS	<0.001
Brachiator	22.59	18.31	16.66	5.14	2.19	**X**	<0.001
Terrestrial quad- climber	106.05	24.50	51.32	31.39	20.55	29.54	**X**

Upper half of plot: p-values, lower half of plot: F-scores.

Stepwise analyses included DA, Tb.N. and ConnD in the final ‘best fit’ solution (thus excluding SMI and Tb.Th.), the results for which are presented here.

### Trabecular Bone Structural Analysis

All bones were scanned on the OMNI-X HD-600 High-Resolution X-ray computed tomography (HRCT) scanner (Varian Medical Systems, Lincolnshire, IL) at the Center for Quantitative Imaging (CQI) at The Pennsylvania State University (PSU). Each specimen was mounted in foam and positioned vertically in the scanner to collect transverse slices through the long bones. Serial cross-sectional scans were collected beginning in the shaft and proceeding proximally to cover the entire femoral or humeral head. For the femur, scans were collected beginning at or near the level of the lesser trochanter. In the humerus, scans were collected beginning just below the surgical neck and progressing proximally. All HRCT scans were collected using source energy settings of either 180 kV/0.11 µA or 150 kV/0.2 µA, between 2800 and 4800 views, and a Feldkamp reconstruction algorithm. The differences in energy settings resulted from a refinement of bone scanning protocols at the PSU CQI over the last 6 years and are unlikely to have an effect on the evaluation of trabecular structure in this study. For each scan, between 41 and 100 slices were collected during each rotation. Voxel sizes ranged between 0.027 and 0.0687 mm depending on the size of the femoral or humeral head. In all cases, the highest-resolution images were obtained given the size of the specimen. The images were reconstructed as 16-bit TIFF grayscale images with a 1024×1024 pixel matrix.

**Figure 3:**
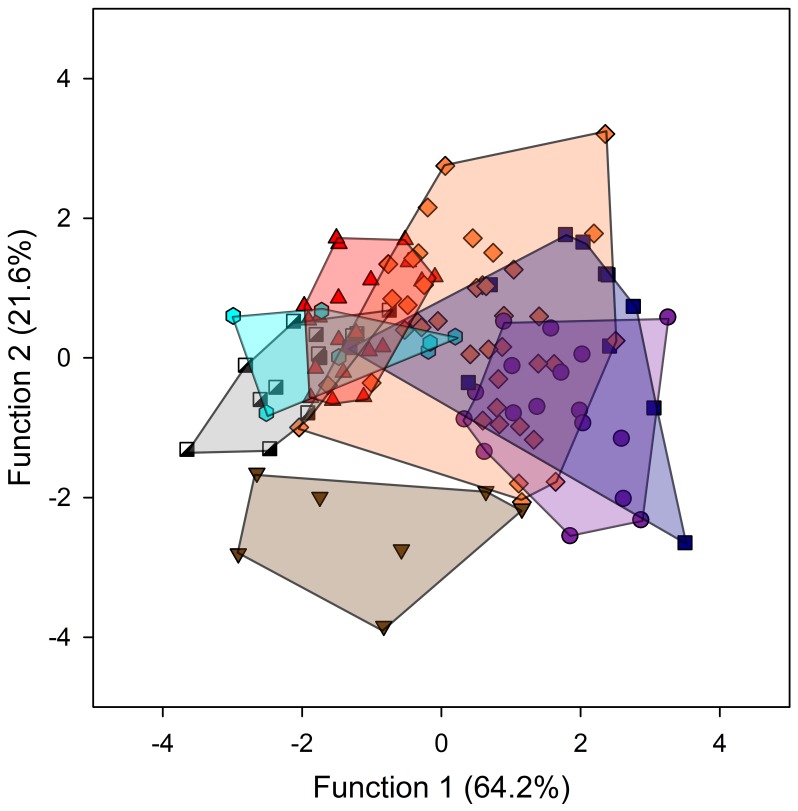
Results of multivariate discriminant function analyses for the humeral head. Symbols as in Figure 2.

**Table 6:** Stepwise discriminant function analyses for locomotor group using seven humeral head trabecular bone variables.

		Predicted group membership							
Locomotor group	n	Biped	Terrestrial quad	Arboreal quad	Arboreal quad-climber	Quadrumanous climber	Brachiator	Terrestrial quad-climber	%correct
Biped	20	14	0	1	1	4	0	0	70
Terrestrial quad	11	0	6	2	1	0	0	2	54.5
Arboreal quad	40	4	6	18	2	2	1	7	45
Arboreal quad-climber	13	1	0	0	11	1	0	0	84.6
Quadrumanous, climber	7	1	0	3	1	2	0	0	28.6
Brachiator	7	0	0	0	1	0	1	1	71.4
Terrestrial quad- climber	17	0	3	3	0	0	11	11	64.7

Classification results (total correct  = 58.3%).

Biped: *Homo*; Terrestrial quadruped: *Papio*; Arboreal quadruped: *Macaca, Trachypithecus*; Arboreal quadruped-climber: *Alouatta*; Quadrumanous climber: *Pongo*; Brachiator: *Symphalangus*; Terrestrial quadruped-climber: *Pan*.

**Table 7:** Pooled within-group correlations (r) between functions and variables.

Variable	Function 1	Function 2
Conn.D	0.423	0.351
SMI	−0.569	0.649
Tb.N	0.867	0.254
Tb.Th	−0.141	−0.061
DA	0.207	0.344

**Table 8:** F-test results between groups (DF  = 3, 106) for stepwise discriminant function analyses for locomotor group using five humeral head trabecular bone variables.

	Biped	Terrestrialquad	Arboreal quad	Arboreal quad-climber	Quadrumanous climber	Brachiator	Terrestrial quad-climber
Biped	**X**	<0.001	<0.001	<0.01	NS	<0.001	<0.001
Terrestrial quad	21.11	**X**	<0.001	<0.001	<0.001	<0.001	<0.001
Arboreal quad	12.06	5.68	**X**	<0.001	<0.01	<0.001	<0.001
Arboreal quad-climber	4.14	29.75	19.90	**X**	<0.05	<0.001	<0.001
Quadrumanous, climber	1.31	12.00	5.00	2.78	**X**	<0.01	<0.001
Brachiator	14.04	23.24	19.07	11.17	4.69	**X**	<0.001
Terrestrial quad- climber	28.94	5.88	10.55	28.92	12.67	20.69	**X**

Upper half of plot: p-values, lower half of plot: F-scores.

Stepwise analyses included DA, Tb.N. and ConnD in the final ‘best fit’ solution (thus excluding SMI and Tb.Th.), the results for which are presented here.

A single cubic volume of interest (VOI) was extracted from the center of the femoral and humeral heads for each individual (Figure 1). The method for determining the size and position of the VOIs using Avizo 6.3 (Visualization Sciences Group, Inc., Burlington MA) is detailed in Ryan and Walker [20] and described briefly here. The articular surface of the femoral or humeral head was defined for each specimen by manually selecting the surface triangles from a three-dimensional isosurface reconstruction. Because a precise division between articular and non-articular regions is not possible to obtain from HRCT data alone (i.e. without other visual and physical clues present on the bones), a conservative approach was taken for all specimens to ensure that non-articular bone was not included in the articular surface selection. The bounding box of the triangulated articular surface shell was defined as the maximum and minimum extents of the articular surface in each of the three orthogonal axes. The center of the bounding box, defined for the purposes of the current analysis as the center of the articular region, was determined by calculating the midpoints of the x, y, and z dimensions of the bounding box.

**Figure 4:**
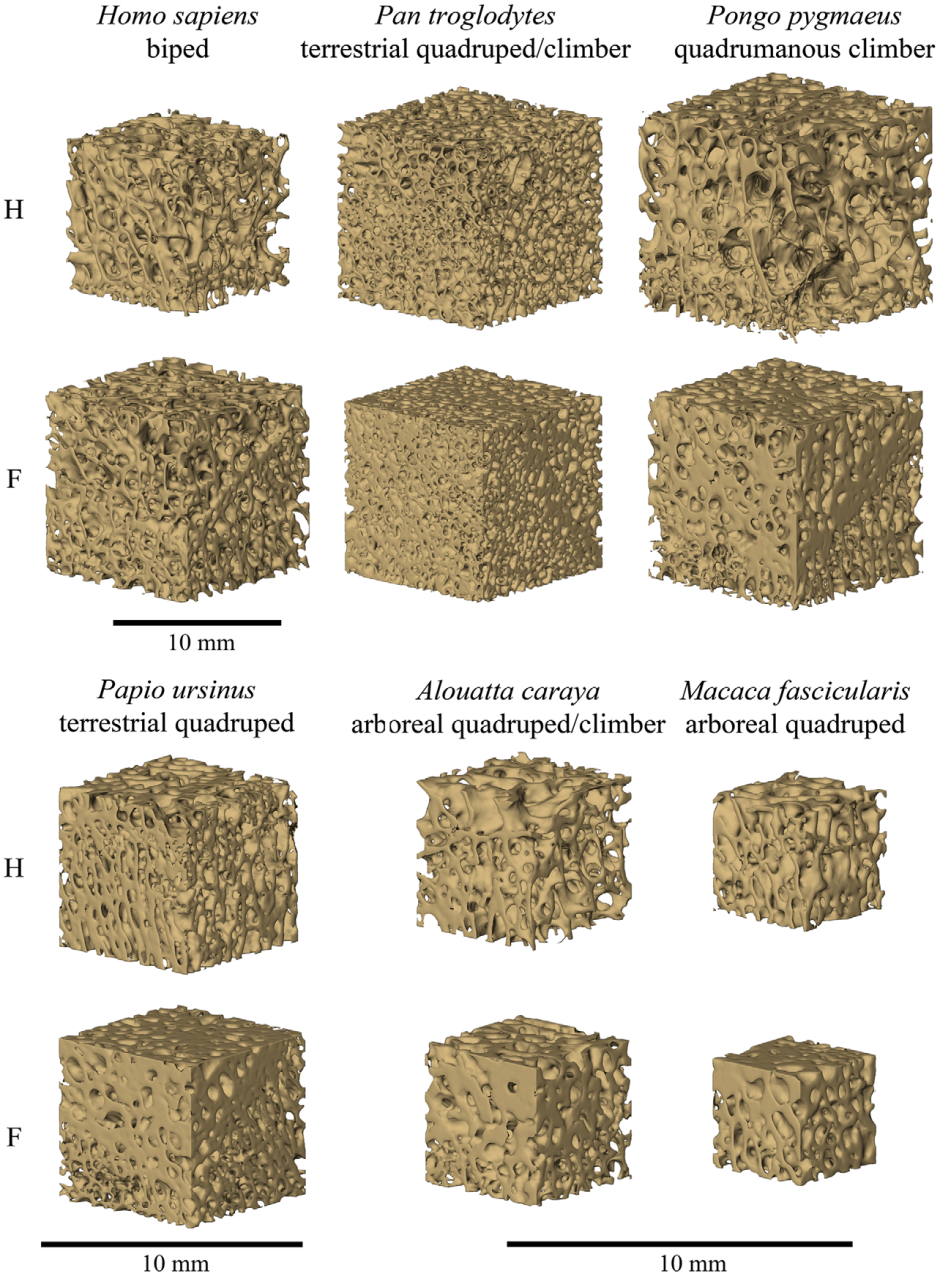
Three-dimensional reconstructions of cubic trabecular bone specimens from the humerus (top) and femur (bottom) from individuals used in the current study. Note variation in trabecular architecture between the femur and humerus, and among taxa.

The center of the VOI was placed at the calculated center of the articular surface bounding box and the edge length of the cube was equal to 1/6 the proximodistal height of the articular surface. This VOI selection protocol ensured that each VOI was positioned homologously (at the center of the joint), and was scaled to the size of the individual joint being analyzed. All measured variables were calculated on a sphere centered within the cubic VOI to avoid corner effects [43]. The VOIs ranged in size from approximately 2.5 to 14 mm in diameter for the humerus, and 2.3 to 15 mm in diameter for the femur. When analyzing trabecular structure in small animals using VOIs scaled by joint size, it is possible that the continuum assumption of Harrigan et al. [44] may not be fully satisfied. Visual inspection of the smallest VOIs used in this study ensured that each one included a minimum of three to five intertrabecular lengths, thereby satisfying this assumption.

The trabecular bone morphometric variables quantified included degree of anisotropy (DA), trabecular number (Tb.N), trabecular thickness (Tb.Th), connectivity density (Conn.D), and structure model index (SMI). Due to strong correlations with other variables and thus the potential to erroneously inflate differences among taxa, some important trabecular bone features typically analyzed in interspecific studies, including bone volume fraction and trabecular spacing, were excluded from the current analysis.

All trabecular bone morphometric analyses were performed using the Scanco Image Processing Language (IPL; Scanco Medical AG, Brüttisellen, Switzerland). The HRCT images were segmented using a threshold value calculated from the iterative segmentation algorithm of Ridler and Calvard [45], [46], based on the grayscale values of the VOI only. This localized segmentation approach ensured appropriate definition of the trabecular bone in the VOI. Segmented data were inspected to ensure appropriate thresholding, and the same threshold value was used for all subsequent morphometric analyses for each individual VOI. Trabecular thickness and number were calculated using model-independent distance transform methods [47]. The structure model index measures the proportion of rod-like and plate-like trabeculae and was calculated following Hildebrand and Ruegsegger [48]. Connectivity density reflects the number of interconnections among trabeculae per unit volume and was calculated following the topological approach of Odgaard and Gunderson [49]. Degree of anisotropy was calculated using the mean intercept length (MIL) method on the three-dimensional volume [50]–[52].

### Resolution Dependency of Trabecular Bone Variables

The spatial resolution (voxel dimensions) of the HRCT datasets used in the current study differs due to the large variation in body sizes, and therefore humeral and femoral head size, across the taxa in the sample. To ensure that this variation in voxel dimensions did not affect the trabecular bone measurements, the resolution dependency of each trabecular bone variable was determined prior to interspecific analysis. The femoral heads of fifteen specimens from five separate species were scanned at an isotropic spatial resolution of 0.014 mm following the same scanning protocols used for the current analysis. The taxa used in this resolution dependency assessment were mostly small-bodied primates due to the need for small limb elements to obtain high resolution. The species included *Galago senegalensis* (n = 5), *Loris tardigradus* (n = 2), *Hapalemur griseus* (n = 1), *Saimiri boliviensis* (n = 2), and *Macaca fascicularis* (n = 5). A cubic VOI was extracted from each specimen following the same VOI sampling procedures used for the current study. Each VOI was then down-sampled to six lower spatial resolutions – isotropic voxel dimensions of 0.02, 0.03, 0.04, 0.05, 0.06, 0.07 mm – using the Resample module in Avizo 6.3 with a Lanczos filter. Trabecular bone structure was quantified at each resolution, for each individual. Least squares linear regression analyses were conducted for each variable to test the relationship with voxel size. The only variable to display a statistically significant relationship (p<0.001) was Tb.Th. While re-sampling to lower spatial resolutions may produce different values than actually re-scanning at those same lower resolutions [53], the ‘corrected’ Tb.Th values obtained in this study are comparable to those found in the literature [11].

A corrected trabecular thickness (Tb.Th.corr) value was calculated to account for the observed voxel size dependency. The Tb.Th calculated from the highest resolution images (voxel size of 0.014 mm) was considered the most accurate value for Tb.Th. For each of the 15 individuals the Tb.Th_0.014_ value was divided by the Tb.Th value calculated at each of the six down-sampled spatial resolutions (i.e., 0.02, 0.03, 0.04, 0.05, 0.06, 0.07 mm). The mean ratio (Tb.Th_0.014_/Tb.Th_x_) at each voxel resolution was then calculated using all 15 individuals included in the resolution dependency analysis sample (*Galago senegalensis, Loris tardigradus, Hapalemur griseus, Saimiri boliviensis,* and *Macaca fascicularis*). Values for these ratios ranged from 0.995 (for the 0.02 mm voxel size) to 0.770 (for the 0.07 mm voxel size). A voxel size correction equation was developed by inputting these six values into a least squares linear regression. The resulting regression equation for Tb.Th (correction factor = −4.4856 * voxel size +1.0805; R^2^ = 0.998; p<0.001) was then applied to the measured Tb.Th values. The resulting Tb.Th.corr values were then used for all subsequent analyses.

### Test for Phylogenetic Signal

Previous tests for a phylogenetic signal in primate long bone structure have demonstrated a significant effect on raw data uncorrected for variation in body size [54]. The influence of phylogeny on trabecular bone structure was tested in this study using the K statistic method [55]. This approach uses generalized least squares to characterize phylogenetic signal given relationships among taxa. The K statistic characterizes phylogenetic signal in a comparative dataset as the ratio between the observed level of phylogenetic covariance in tip data and the expected level of covariance under a Brownian motion model of character evolution. As defined by Blomberg et al. [55], a K statistic less than 1 indicates that close relatives resemble each other less than expected. Conversely, a K value greater than 1 indicates that closely related taxa are more similar than expected under Brownian motion. While 8 taxa are adequate for the calculation of K [56], at least 20 taxa are required to calculate the statistical significance of a phylogenetic signal using randomization tests [55]. As a result, statistical differences were not calculated here.

Phylogenetic signal was calculated for each trabecular bone variable as follows. The log_10_ transformed values for each trabecular bone variable for each individual were regressed against log_10_ transformed estimates of body mass for each individual (see below) using ordinary least squares regression. The unstandardized residuals from each of these regression analyses were calculated and represented a body mass-standardized variable. The specialized Matlab code PHYSIG was used to calculate the strength of the phylogenetic signal for the species mean of each body mass-standardized variable [55]. The phylogenetic tree for the 8 taxa (Information S1) used estimated divergence dates as branch lengths and was based on published phylogenetic analyses [57]–[62]. For each variable, K is much less than 1, indicating less phylogenetic signal than expected under a Brownian motion model of character evolution (Table 2). While these tests do not necessarily indicate that phylogenetic signal is completely absent in trabecular bone structure in these taxa, the results strongly suggest that size-corrected variables are unlikely to contain a strong phylogenetic signal, and thus are acceptable for use in the multivariate analyses conducted in this study.

### Discriminant Function Analyses

Discriminant function analyses were used to test the utility of the femoral and humeral head trabecular bone variables for differentiating anthropoid taxa into locomotor categories. Recent work on mammalian trabecular bone has demonstrated an allometric scaling effect on trabecular structural features [63]. To correct for the influence of body size, log_10_ transformed values for each trabecular bone variable were regressed against a log_10_ transformed estimate of body mass for each individual, using ordinary least squares linear regression. While using estimates of body mass based on regression equations introduces error, the non-isometric scaling of femoral head size with body size in *Homo sapiens* [64], [65] precludes the use of femoral head breadth as a proxy for body mass in this sample. Body mass for each individual was estimated from femoral head dimensions using equations taken from the literature [66]–[68] and derived from analyses of the most appropriate taxonomic group (Table 1). Femoral head anteroposterior breadth, mediolateral breadth, and superoinferior height were measured to the nearest hundredth of a millimeter using digital calipers.

For all 115 individuals in the sample, unstandardized residuals calculated for the five trabecular bone variables were input into discriminant function analyses. Analyses were run separately for the humeral and femoral variables. To calculate structure matrices and locomotor group membership, unstandardized residuals were entered together and locomotor category was used as the grouping variable. To calculate the f-statistic for between locomotor group differences, discriminant analyses were re-run using a stepwise approach. All statistical analyses were performed in SPSS 18.0. For all statistical tests, null hypotheses were rejected for P-values less than 0.05.

## Results

The stepwise analysis of femoral head trabecular bone morphology for the complete sample generated two significant discriminant functions (p*<*0.001) that account for 68.5% and 23.1% of the variance, respectively (Figure 2). These functions correctly classify 85 of the 115 specimens (73.9%) into their assigned locomotor categories. The percentage of correct classifications varies by group (Table 3). Function 1 shows the strongest correlation with Tb.N and SMI, while Function 2 correlates most strongly with Tb.N and DA (Table 4). An F-test of among-group separations is highly significant (*P<*0.001, Table 5) for virtually all locomotor groups. The exception to this finding is the comparison of terrestrial versus arboreal quadrupeds and quadrumanous-climbers versus both arboreal quadruped-climbers and brachiators. This stepwise F-test initially included all five Tb variables. However, because SMI and Tb.Th were not included in the ‘best fit’ final solution, between group differences are based on three variables, DA, Tb.N and Conn.D.

Function 1 differentiates terrestrial quadrupedal-climbers (*Pan*) on one end of a continuum from bipeds (*Homo*) on the other. On this continuum, all remaining quadrupeds (*Alouatta, Macaca, Papio, Trachypithecus*) cluster together with quadrumanous climbers (*Pongo)* and brachiators (*Symphalangus)*. Overlap exists between bipeds (*Homo*) and a few arboreally quadrupedal *(Macaca* and *Trachypithecus*) individuals. Function 2 separates brachiators (*Symphalangus*), arboreal quadrupedal-climbers (*Alouatta*) and, to a lesser degree, quadrumanous climbers (*Pongo*), from terrestrial quadrupeds (*Papio*). Arboreal quadrupeds (*Macaca, Trachypithecus*), bipeds (*Homo*) and terrestrial quadrupedal-climbers (*Pan*) fall in between these more extreme positions and overlap with all other groups.

The analysis of trabecular morphology of the proximal humerus also generated two significant discriminant functions (p*<*0.001) that accounted for 64.2% and 21.6% of the variance, respectively (Figure 3). These functions correctly classify 67 of the 115 specimens (58.3%) into their assigned locomotor groups, but the percentage of correct classifications varies by group (Table 6). Function 1 is strongly correlated with Tb.N and SMI, while Function 2 correlate with SMI and Conn.D (Table 7). Function 1 differentiates terrestrial quadrupeds and terrestrial quadrupedal-climbers (*Papio,* and *Pan*, respectively) from bipeds, quadrumanous climbers and arboreal quadrupedal-climbers (*Homo*, *Pongo, Alouatta*, respectively). Arboreal quadrupeds (*Macaca* and *Trachypithecus*) and brachiators (*Symphalangus*) overlap with all other groups. In contrast, for Function 2 brachiators (*Symphalangus*) are virtual outliers, separated from all other groups that are tightly clustered. Additionally, an F-test of between-group separations is significant among all locomotor groups (p*<*0.001, Table 8) other than bipeds and quadrumanous climbers. Similar to the outcome of the stepwise F-test conducted for femoral head trabecular analyses, the humeral head analysis initially included all five Tb variables. However, because SMI and Tb.Th are not included in the ‘best fit’ final solution, between group differences are based on only three variables, DA, Tb.N and Conn.D.

## Discussion

Two hypotheses were tested in this study. Groups employing more distinct locomotor patterns were expected to have significantly divergent trabecular architecture, while groups with more similar locomotor patterns were expected to display more similar trabecular structure. The results reveal significant differences in trabecular architecture among locomotor categories. Nevertheless, overlap in trabecular morphology was found among individuals from both more (i.e. arboreal quadruped vs. arboreal quadruped-climber) and less distinct (i.e. brachiator vs. arboreal quadrupeds) locomotor behavioral groups. Because the forelimbs of primates generally experience lower vertical peak reaction forces during locomotion, it was hypothesized that humeral head trabecular structure would not distinguish locomotor groups as effectively as would trabecular structure from the femoral head. The results appear to support this hypothesis. Although significant differences exist among locomotor categories for both humeral and femoral head trabecular morphology, overlap among groups is more apparent in the proximal humerus.

The results of the current study suggest that when multiple trabecular bone variables are considered together as a functioning morphological suite, a locomotor signal may be detectable in the anthropoid femoral head, and less obviously in the humeral head. This finding may prove useful for reconstructions of locomotor behavior in the fossil record. Generally, the percentage of correct predictions derived from femoral head trabecular bone features (73.9%) indicates bone structural differences associated with generalized locomotor behaviors. Locomotor group predictions derived from the humeral head trabecular architecture were less accurate (58.3%).

The challenge stemming from the current study is to determine the mechanical and functional significance of the suite of morphological traits – Tb.N, Conn.D, DA, and SMI – that differentiates these locomotor groups. These features are intriguing due to their established relationships to the ultimate strength and elastic properties of trabecular bone [1]–[9]. The results from the current analysis suggest that multivariate analyses differentiate locomotor groups based on a combination of mechanically-relevant morphological features. This correspondence between a) variables that appear to delineate locomotor behavioral differences and, b) variables found to be significant in determining the mechanical behavior of trabecular bone (pre- and post-yield), suggests a locomotion-specific functional signal in the subarticular trabecular bone architecture of the femur and, less obviously, the humerus.

In spite of this apparent correspondence between the structural and mechanical properties of anthropoid trabecular bone, constructing direct links between trabecular bone variation and specific loading conditions for each locomotor mode is more problematic. It is apparent from these results that in the femoral head, bipeds (*Homo*) display unique trabecular bone characteristics that include a relatively low number of thin, concave plate-like, trabeculae that are highly anisotropic (Figure 4). This unique suite of traits sets them apart from almost all other taxa and, coupled with the low bone volume fraction [32], suggests relatively low tissue elasticity [1], [3], [4], [6]. The asymmetric placement of trabecular bone in the femoral neck of humans, a phenomenon termed trabecular eccentricity, has been identified as a stress-reducing mechanism that reflects bone adaptation to applied loads [69]. Trabecular eccentricity and the femoral head bone architecture identified in this study suggest unique solutions to mechanical demands in the human proximal femur.

In direct contrast to the patterns seen in *Homo*, terrestrial quadruped-climbers (*Pan*) display relatively numerous, thick, highly concave trabeculae that form a dense, isotropic bone structure (Figure 4). These results suggest a fundamentally different trabecular bone architecture in the femur of Pan and, consequently, different mechanical properties. The femoral head trabecular structure of the remaining locomotor groups falls between the two ends of the continuum occupied by *Pan* and *Homo* (Figure 4). The terrestrial and arboreal quadrupedal specimens (*Papio* and *Macaca*, respectively) are associated with a more densely packed, concave, anisotropic trabecular bone structure. Brachiating (*Symphalangus*), quadrumanous climbing (*Pongo*), and arboreal quadruped-climbing (*Alouatta*) taxa have fewer, relatively isotropic trabeculae.

The microstructure of humeral head trabeculae presents a tripartite, though somewhat overlapping, separation of the locomotor groups. The brachiating locomotor pattern unique to *Symphalangus* corresponds with trabecular architecture that is relatively gracile, characterized by few, closed plate-like trabeculae that are loosely packed to form a relatively isotropic structure. In contrast, a more robust architecture is associated with the arboreal (*Macaca, Trachypithecus*) and terrestrial (*Papio*) quadrupedal and terrestrial quadrupedal-climbing (*Pan*) taxa, defined by numerous closed, concave trabeculae that form a densely packed relatively anisotropic structure (Figure 4). Finally, clustered tightly are the bipedal (*Homo*), quadrumanous climbing (*Pongo*), and arboreal quadruped-climbing (*Alouatta*) taxa, all of whom display a low number of trabeculae that are thickened and plate-like, and not highly connected. Along Function 1 *Homo*, *Pongo, Alouatta,* and *Symphalangus* overlap significantly, suggesting broadly similar bone structure, perhaps reflecting lower magnitude loading on the proximal humerus as compared to quadrupedal catarrhines.

Interestingly, bipeds and quadrumanous climbers present relatively similar humeral head trabecular bone morphology. The differences between the relative (un)loading of the forelimbs during terrestrial bipedal locomotion in *Homo* and forelimb loading during quadrumanous climbing in *Pongo* are presumably quite pronounced. The finding of microarchitectural similarities in the humeral head of these two taxa is therefore surprising. This result is interesting given the proposal that *Pongo*-like bipedal clambering may represent the primitive great ape locomotor pattern [70]–[72], but it is not immediately clear what drives these structural similarities.

Trabecular bone architecture is the product of both genetic and environmental influences [10], [73], [74], [75]. The sensitivity of bone to mechanical signals, and the consequent functional importance of bone structural variation, is well established [76]. Environmental influences including locomotor, dietary, and manipulative behaviors can shape trabecular form throughout an individual’s lifetime. Whether the interspecific differences in trabecular bone architecture described in this study are the result of selective or environmental forces, or a combination of both, remains unclear. While deciphering these influences is not the primary aim of this study, it is worth noting that the correspondence between trabecular architecture and distinct locomotor patterns in anthropoids may reflect selection over multiple generations and/or adaptation over the course of the lifetime of an individual.

One of the limitations of the present study is the use of relatively broad locomotor categories, each composed of a single taxon or a small number of taxa. As a result, structural differences across locomotor groups may reflect species-specific attributes rather than exclusively locomotor behavioral differences. Although it has been demonstrated that little or no phylogenetic signal exists in the morphological variables used in this study, phylogenetic heritage may nevertheless still be influencing the results. Given locomotor behavioral variation in extant primates, the number of potential species available for inclusion within certain locomotor categories is necessarily restricted (i.e. bipeds and quadrumanous climbers). Future studies that include a greater number of taxa within each locomotor category, where possible, will be better able to identify those characteristics of trabecular bone structural variation capable of differentiating among locomotor groups. It is also possible that the relatively coarse locomotor categories used in this study mask species-specific variation in locomotor behaviors. The ability to use trabecular bone architecture of the hip and shoulder joints to reconstruct locomotor behaviors in extinct primate taxa will depend on the continued refinement of the methods presented here and the determination of the relative importance of the phylogenetic, developmental, and functional components of bone architecture.

The challenge in future comparative trabecular bone analyses will be to elucidate further the nature of the behavior-specific osteological signal. Further analysis of ontogenetic changes in trabecular architecture will shed light on aspects of trabecular bone morphology that are mechanically mediated and functionally relevant [11], [13], [77]. Similarly, continued study of the interrelationships between trabecular bone architectural and mechanical variation will further delineate the functional significance of structural variation in the mammalian postcranial skeleton. Treating trabecular bone morphology as a suite of interrelated traits takes a step towards a Systems Biology approach, which considers complex interactions between components of a biological organism. Integrating trabecular bone architecture into analyses and models of whole bone structure and function will help define the interaction between the micro- and macro-scale structural adaptations of bone and how these interactions influence the function and behavior of the entire skeletal element.

### Conclusions

By partitioning trabecular morphology into its component parts, previous studies may have inadvertently precluded identification of a more robust ‘locomotor signal’ in the primate locomotor skeleton. The findings of the present study suggest that morphological patterns reflective of adaptation to habitual locomotor mode may be identified in anthropoid primates through a combined analysis of multiple trabecular bone variables together. Applied to analyses of fossil primate skeletal remains, this approach might be a useful tool for inferring habitual locomotor patterns. Nevertheless, caution is advisable. While the eight taxa assessed here were reasonably successfully partitioned by general locomotor mode, such categorization does not encompass the variety of behaviors utilized by individuals or species as a whole. If a comparable approach were used to infer locomotor patterns from primate fossil remains, only general descriptions would be appropriate.

## Supporting Information

Information S1 **Phylogenetic tree used in the current study in Newick format.**
(DOCX)
