# Supplementary material for: Unique Suites of Trabecular Bone Features Characterize Locomotor Behavior in Human and Non-Human Anthropoid Primates
Source: PLoS One. 2012 Jul 18;7(7):e41037. doi: 10.1371/journal.pone.0041037 (PMC3399801; doi:10.1371/journal.pone.0041037)
Supplement: Information S1 — Phylogenetic tree used in the current study in Newick format. (DOCX) [file pone.0041037.s001.docx]

**Information S1.** Phylogenetic tree used in the current study in Newick format.

((((Pongo_pygmaeus: 11.3, (Homo_sapiens: 5.4,Pan_troglodytes: 5.4):5.9): 3.7,Symphalangus_syndactylus: 15): 15.5, ((Macaca_fascicularis: 9.8,Papio_sp:9.8):6.2,Trachypithecus_cristata: 16):14.5):12.4,Alouatta_caraya:42.9);
